# COME: contrastive mapping learning for spatial reconstruction of single-cell RNA sequencing data

**DOI:** 10.1093/bioinformatics/btaf083

**Published:** 2025-02-24

**Authors:** Xindian Wei, Tianyi Chen, Xibiao Wang, Wenjun Shen, Cheng Liu, Si Wu, Hau-San Wong

**Affiliations:** Department of Computer Science, City University of Hong Kong, Kowloon 999077, Hong Kong; Department of Computer Science, City University of Hong Kong, Kowloon 999077, Hong Kong; Department of Computer Science, Shantou University, Shantou 515063, China; Department of Bioinformatics, Shantou University Medical College, Shantou 515041, China; Department of Computer Science, Shantou University, Shantou 515063, China; Department of Computer Science and Engineering, South China University of Technology, Guangzhou, Guangdong 510006, China; Department of Computer Science, City University of Hong Kong, Kowloon 999077, Hong Kong

## Abstract

**Motivation:**

Single-cell RNA sequencing (scRNA-seq) enables high-throughput transcriptomic profiling at single-cell resolution. The inherent spatial location is crucial for understanding how single cells orchestrate multicellular functions and drive diseases. However, spatial information is often lost during tissue dissociation. Spatial transcriptomic (ST) technologies can provide precise spatial gene expression atlas, while their practicality is constrained by the number of genes they can assay or the associated costs at a larger scale and the fine-grained cell-type annotation. By transferring knowledge between scRNA-seq and ST data through cell correspondence learning, it is possible to recover the spatial properties inherent in scRNA-seq datasets.

**Results:**

In this study, we introduce COME, a COntrastive Mapping lEarning approach that learns mapping between ST and scRNA-seq data to recover the spatial information of scRNA-seq data. Extensive experiments demonstrate that the proposed COME method effectively captures precise cell-spot relationships and outperforms previous methods in recovering spatial location for scRNA-seq data. More importantly, our method is capable of precisely identifying biologically meaningful information within the data, such as the spatial structure of missing genes, spatial hierarchical patterns, and the cell-type compositions for each spot. These results indicate that the proposed COME method can help to understand the heterogeneity and activities among cells within tissue environments.

**Availability and implementation:**

The COME is freely available in GitHub (https://github.com/cindyway/COME)

## 1 Introduction

The advent of high-throughput single-cell RNA sequencing (scRNA-seq) technologies has enabled gene expression profiling in thousands of individual cells. This has revolutionized our ability to study cellular processes with single-cell resolution. However, a notable limitation of scRNA-seq protocols is that they necessitate the dissociation of tissues, resulting in the loss of crucial information concerning the native spatial context and cellular positioning. In contrast, this spatial information is essential for comprehending biological mechanisms such as the localization of cell types, intercellular communication, and cellular trajectories.

Spatially resolved transcriptomics can bridge the gap between cell molecular identities and organ-level tissue organization. Spatial transcriptomic (ST) technologies record both the spatial position and gene expression levels of spots on a tissue. However, imaging-based ST technologies (Williams *et al.* 2022), such as including multiplexed error-robust fluorescence *in situ* hybridization (MERFISH) (Chen *et al.* 2015), single-molecule FISH (smFISH) (Codeluppi *et al.* 2018), spatially resolved transcript amplicon readout mapping (STARmap) (Wang *et al.* 2018), and sequential fluorescence *in situ* hybridization (seqFISH+) (Eng *et al.* 2019), can be equipment-intensive, time-consuming and have limited per-spot gene detection capacity if they offer higher spatial resolutions. In contrast to ST, scRNA-seq can capture a significantly higher number of genes for each cell, and it is relatively easier to obtain a larger number of scRNA-seq datasets (Longo *et al.* 2021). While sequence-based ST, such as 10× Visium, enables the detection of numerous gene expressions at each spot, it lacks single-cell resolution and cell-type annotations characteristic of scRNA-seq data. Therefore, establishing cell correspondences between scRNA-seq and ST datasets enables the possibility of reconstructing the spatial information for scRNA-seq cells. This enables the transfer of knowledge across the two modalities, allowing for the retrieval of the inherent spatial characteristics of single-cell data.

Early methods for cell correspondence learning between scRNA-seq and ST data are mainly based on correlating gene expression patterns across scRNA-seq and ST modalities. Specifically, DistMap (Karaiskos *et al.* 2017) calculates Matthews correlation coefficients (MCCs) between cells and ranks the MCCs to assign spatial positions. Peng *et al.* (2016) used Spearman rank correlation to match cells based on co-expression similarity. Achim *et al.* (2015) developed a scoring system to rank the likelihood of cell correspondences based on expression pattern similarity and distance between putative spatial locations. These basic correlation or similarity-based approaches are prone to noise and often produce ambiguous mappings due to the high dimensionality of single-cell expression data. Additionally, methods like Seurat V3 (Stuart *et al.* 2019), Harmony (Korsunsky *et al.* 2019), LIGER (Welch *et al.* 2019), and SpaGE (Abdelaal *et al.* 2020) utilize dimension reduction techniques to embed scRNA-seq and ST data into a shared low-dimensional space for cell correspondence matching. These approaches face challenges in information loss and interpretability when projecting to a low-dimensional representation.

Recently, SpaOTsc (Cang and Nie 2020) and novoSpaRc (Moriel *et al.* 2021) construct optimal transport (OT) cost matrix using MCCs and K-Nearest Neighbors (KNNs), respectively, to measure cell–cell similarity. These approaches of outputting the mapping matrix enable various spatial associations with scRNA-seq data, facilitating tasks such as gene imputation in imaging-based ST and spot deconvolution in sequence-based ST. Moreover, deep learning models have been applied to learn nonlinear representations for discovering cell mapping between scRNA-seq and ST data. For example, Tangram (Biancalani *et al.* 2021) introduces a pairwise cosine similarity scoring method as an objective function to learn a mapping matrix. Uniport (Cao *et al.* 2022) integrates the OT distance matrix into a latent space of the Variational Autoencoder (Kingma and Welling 2014) to align two datasets but does require significant memory space for the optimization process. Meanwhile, GraphST (Long *et al.* 2023) presents a KNNs graph-based neural network approach incorporating two datasets with a projection matrix. In addition to cell correspondence learning, several methods such as gene imputation and spot deconvolution are designed for one-way information transfer from scRNA-seq data to ST. For example, in the context of imaging-based ST data, gimVI (Lopez *et al.* 2019) is a probabilistic generative model that imputes missing gene values by referencing scRNA-seq data. On the other hand, spot deconvolution methods, such as SPOTlight (Elosua-Bayes *et al.* 2021), spatialDWLS (Dong and Yuan 2021), Cell2location (Kleshchevnikov *et al.* 2022), SpatialDecon (Danaher *et al.* 2022), STRIDE (Sun *et al.* 2022), CARD (Ma and Zhou 2022), SD^2^ (Li *et al.* 2022b), RCTD (Cable *et al.* 2022), stVAE (Li *et al.* 2023), and DOT (Rahimi *et al.* 2024), focus on leveraging sequencing-based ST to transfer cell-type annotations from scRNA-seq data to ST.

In this work, we develop a novel mapping approach that captures relationships between ST data and scRNA-seq data for the spatial reconstruction of scRNA-seq profiles. While existing methods such as SpaOTsc, novoSpaRc, Tangram, and GraphST utilize spatial mapping for the reconstruction of scRNA-seq data, they have distinct focuses and limitations. For instance, GraphST and novoSpaRc incorporate positional information and emphasize local associations by optimizing neighbor selection. SpaOTsc also leverages spatial data but assumes cell-to-cell relationships using metrics like MCCs. However, most current approaches focus primarily on modeling local relationships, either through neighbor selection or predefined cell-to-cell interactions, and fail to automatically learn both local and global relationships through cross-representation learning. This leads to inconsistent spatial mappings. Furthermore, these methods overlook the potential of incorporating scRNA-seq cell-type information, which is crucial for more accurate learning. Cells with similar functions typically display correlated expression patterns, as noted in Harris *et al.* (2021). Therefore, an scRNA-seq cell can often be linked to multiple spatial locations sharing similar expression profiles. Integrating cell-type spatial dependencies is critical for capturing biologically meaningful interactions between cell types across tissues.

To address these limitations, our approach incorporates a contrastive learning framework that includes cell-type contrastive learning for scRNA-seq feature representation and inter-contrastive learning for both scRNA-seq and ST data. Specifically, we propose an adaptable and trainable mapping network that seamlessly integrates a structural similarity regularization loss for network optimization, through which meaningful cellular latent features can be effectively encoded to learn precise cell correspondences mapping. Furthermore, we develop a contrastive learning approach to exploit more meaningful latent feature representations across two modalities. More specifically, the contrastive learning framework comprises cell-type contrastive learning for feature representation learning of scRNA-seq data, and inter-contrastive learning for feature representation learning of both scRNA-seq and ST datasets. By leveraging the available cell-type information of scRNA-seq data and the learned mapping matrix, we aim to achieve spatial awareness and distinguish between similar cell types within the latent feature representations of both modalities. Extensive experiments on three different biological systems with different spatial reconstruction tasks have demonstrated that our method surpasses state-of-the-art methods across various metrics. The learned mapping results regarding biological insights can further verify the accurate localization of biologically meaningful cell types, extend gene spatial patterns of scRNA-seq data, and deconvolve cell-type composition. This approach allows for a more extensive knowledge of cellular interactions and functions, providing valuable insights into the spatial structure of cells in multicellular systems.

## 2 Materials and methods

**Figure 1.**
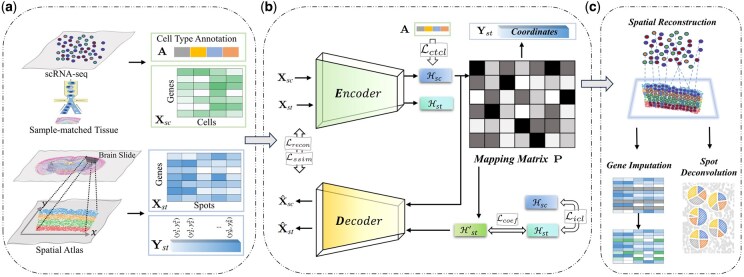
The pipeline of the proposed method. (a) The processing of data. (b) The network architecture of COME. The network takes ST data $X_{st}$ and scRNA-seq data $X_{sc}$ as input. To encode meaningful cellular features, the input data are encoded and decoded to latent representations $\left(H,\hat{X}\right)$. Concurrently, $H_{sc}$ is input to a mapping matrix P for learning cell-spot gene expression relationships with the SSIM regularization, producing an improved target matrix. Optimization is applied to P via inter-contrastive learning with cell type A. By matching P to $Y_{st}$, spatial locations are predicted for $X_{sc}$. (c) The outputs P of COME can be utilized for gene imputation and spot deconvolution.

In this section, we provide a detailed description of our proposed method, which seeks to spatially reconstruct scRNA-seq data by assigning a single cell to the corresponding coordinates of spatial reference. The pipeline of our approach for spatially mapping scRNA-seq data is illustrated in Fig. 1.

### 2.1 Problem formulation

The input data consists of a ST dataset $X_{st}\in R^{N\times P}$ and a scRNA-seq dataset $X_{sc}\in R^{M\times Q}$, where *N, P* are the number of cells and genes for the ST data and *M, Q for the scRNA-seq data.*

Typically, Q≫P, as scRNA-seq can capture a lot of gene expressions without spatial information. In practice, we can align the two modalities based on these shared genes by selecting a subset of *K* overlapping genes in the ST and scRNA-seq data. Therefore, by prepossessing these *K* overlapping genes, the data can be represented as ST data $X_{st}\in R^{N\times K}$ and scRNA-seq data $X_{sc}\in R^{M\times K}$. In this setting, $Y_{st}\in R^{N\times 2}$ is the spatial coordinates matrix of ST. The key to predicting the spatial information $Y_{sc}$ for the scRNA-seq data is to learn the cell-to-spot mapping $P\in R^{M\times N}$ between the ST and scRNA-seq data, and then predict $Y_{sc}$ with the learned mapping P. The mapping probability matrix P in our method serves dual purposes: predicting spatial information for scRNA-seq cells and performing cell spot deconvolution in ST data. For spatial prediction of scRNA-seq data, our method assigns spatial information to scRNA-seq cells by leveraging the closest ST data. Specifically, using the probability matrix P, we estimate the spatial locations based on ST data, which then acts as the predicted spatial coordinates for corresponding scRNA-seq cells. In addition, for spot deconvolution in ST data analysis, where the goal is to infer cell types or mixtures of cell types at each spot, the probability matrix P offers a flexible framework. P allows for the prediction of mixed cell types at each spatial location, enabling more accurate modeling of complex cellular compositions in ST data.

### 2.2 Cell correspondence learning

In the proposed model, a shared autoencoder is initially employed to derive latent representations for both the ST data $X_{st}$ and the scRNA-seq data $X_{sc}$. To capture cell-spot relationships between modalities, the reconstructed spatial data ${\hat{X}}_{st}$ is obtained by decoding the latent code $H_{sc}$ from the scRNA-seq data encoder $E(X_{sc})$. A coefficient layer is also introduced to learn cell-spot matching by mapping from scRNA-seq to the spatial domain. This coefficient matrix $C\in R^{M\times N}$ takes $H_{sc}$ as input and predicts corresponding spatial data via $H_{st}^{\prime }=C^{⊤}H_{sc}$. The adaptive matrix C captures the raw association strength without relying on predetermined relationships between cells and spots. To better capture coefficient patterns during training, the process can be formulated as follows:

(1) $${\hat{X}}_{st}=D(C^{⊤}E(X_{sc})),$$

 

(2) $${\hat{X}}_{sc}=D(E(X_{sc})).$$

The corresponding reconstruction loss function can be defined as follows:

(3) $$L_{\mathrm{recon}}=||X_{st}-{\hat{X}}_{st}|{|}_{2}^{2}+||X_{sc}-{\hat{X}}_{sc}|{|}_{2}^{2},$$

where $||\cdot |{|}_{2}^{2}$ calculates mean squared error. We formulate the regularized coefficient loss term as:

(4) $$L_{\mathrm{coef}}=||H_{st}-H_{st}^{\prime }|{|}_{2}^{2}+\lambda ||C|{|}_{2}^{2},$$

where λ represents the weighting factors used to balance the relative importance of the regularization terms. Norm regularization $||C|{|}_{2}^{2}$ is applied to the mapping matrix to enforce matrix normalization. The mapping matrix P is computed by applying softmax (Goodfellow 2016) to the coefficient matrix C over columns:

(5) $$P_{ij}=\frac{\;\text{exp}(C_{ij})}{\sum_{k}exp(C_{ik})}$$

This normalization enhances the interpretability of the mapping matrix P, where each row of P represents the probability of each scRNA-seq cell being located at the corresponding spatial spot.

The coefficient matrix C is of full size (M×N), not batch-wise, allowing it to learn cell spatial patterns at the entire tissue level. Consequently, the mapping matrix P captures the associations within the latent space and between ST data and scRNA-seq data.

To highlight the significance of preserving structure with the ground truth matrix, we introduce a structural similarity regularization term $L_{\mathrm{ssim}}$ to measure the consistency of structures between matrices. This loss function is particularly sensitive to spatial pixel variations, making it ideal for assessing gene expression patterns in a global context. Given that the reconstructed spatial gene expression ${\hat{X}}_{st}$ is derived from the latent features $H_{sc}$ (i.e. ${\hat{X}}_{st}=D(P^{⊤}H_{sc})$), $L_{\mathrm{ssim}}$ extends optimization beyond the latent space. To achieve this, we construct another reconstruction matrix ${\tilde{X}}_{st}=P^{⊤}X_{sc}$ directly from the original gene expression data, and then apply $L_{\mathrm{ssim}}$ to minimize the structural dissimilarity between the predicted and actual spatial gene expression in the global feature space. The specific form of this structural similarity measure can be expressed as follows:

(6) $$\mathrm{Sim}(X_{st},{\tilde{X}}_{st})=\frac{\left(2\mu_{X_{st}}\mu_{{\tilde{X}}_{st}}+Z_{1})(2Cov(X_{st},{\tilde{X}}_{st})+Z_{2}\right)}{\left(\mu_{X_{st}}^{2}+\mu_{{\tilde{X}}_{st}}^{2}+Z_{1})(\sigma_{X_{st}}^{2}+\sigma_{{\tilde{X}}_{st}}^{2}+Z_{2}\right)},$$

where Cov(·,·) calculates the covariance between two entities. μ and σ denote the mean and standard deviation, respectively. The terms $Z_{1}$ and $Z_{2}$ denote constant normalization terms, both derived by multiplying a constant with the maximum value of the input. Specifically, this can be expressed as: $Z_{1}=a_{1}\cdot \max(X_{st},{\tilde{X}}_{st})$ and $Z_{2}=a_{2}\cdot \max(X_{st},{\tilde{X}}_{st})$. (In this context, we empirically set the constant values $a_{1}=0.01$ and $a_{2}=0.03$.) We propose the following structural similarity loss as follows:

(7) $$L_{\mathrm{ssim}}=1-\mathrm{Sim}(X_{st},{\tilde{X}}_{st})$$

where the structural similarity Sim(·,·) ranges from 0 to 1, where higher values denote greater similarity.

### 2.3 Contrastive learning module

Moreover, we aim to develop a contrastive learning approach to uncover more meaningful latent feature representations across two modalities. By leveraging the available cell-type information and the mapping matrix, we seek to achieve spatial awareness and distinguish between similar cell types within the latent feature representations of both modalities.

Specifically, for the scRNA-seq data, cell samples within the same cell type should exhibit similar feature representations. Therefore, we develop a cell-type contrastive learning approach for the scRNA-seq data by leveraging the available cell-type information. In this intra cell-type contrastive learning, cell pairs of the same type are considered positive, whereas those of different types are considered negative. The cell-type labels are defined as y. The intra-contrastive learning can be formulated as follows:

(8) $$L_{\mathrm{ctcl}}=-\frac{1}{M}\sum_{i=1}^{M}log\frac{\sum_{y_{i}=y_{j}}f(H_{sc}^{i},H_{sc}^{j})}{\sum_{y_{j}\neq y_{i}}f(H_{sc}^{i},H_{sc}^{j})}$$

where $f(a,b)=e^{\theta (a,b)/\tau }$ and inner product $\theta (a,b)=a^{T}b/||a||||b||$ is used to calculate the pair’s similarity, and τ is a temperature parameter. This formulation allows us to push cell i and cell j closer together when they belong to the same cell type. Moreover, latent feature representations of scRNA-seq and ST are expected to be similar. To achieve this, we develop an inter-contrastive learning approach across the ST and scRNA-seq datasets by leveraging the learned mapping matrix P. Specifically, for the scRNA-seq feature representation $H_{sc}^{i}$, the associated positive sample in the ST data is $H_{st}^{j}$, which has the maximum probability connection of two samples in the learned mapping matrix P. Thus, the inter-contrastive loss function can be defined as:

(9) $$L_{\mathrm{icl}}=-\frac{1}{M}\sum_{i=1}^{M}log\frac{f(H_{sc}^{i},H_{st}^{j})}{\sum_{k\neq j}f(H_{sc}^{i},H_{st}^{k})}$$

Overall, the unified contrastive loss function can be expressed as:

(10) $$L_{\mathrm{cst}}=L_{\mathrm{icl}}+L_{\mathrm{ctcl}}$$

We utilize the discrete version of the loss to optimize the network. By incorporating the aforementioned regularization methods, the optimization formulation is expressed as follows:

(11) $$\underset{\theta_{E},\theta_{D},C}{\min}L_{\mathrm{recon}}+\alpha_{1}L_{\mathrm{coef}}+\alpha_{2}L_{\mathrm{ssim}}+\alpha_{3}L_{\mathrm{cst}}$$

where $\alpha_{1}$, $\alpha_{2}$, and $\alpha_{3}$ are weighting factors for the loss terms. The term $L_{\mathrm{recon}}$ ensures that the autoencoder extracts informative features from the input gene expression. Due to the global structure constraint term $L_{\mathrm{ssim}}$, we can ensure that the variation between genes across the entire tissue is accounted for. In addition, the term $L_{\mathrm{coef}}$ is useful for modeling the cell spatial characteristic across cell modalities, and the term $L_{\mathrm{cst}}$ facilitates accurate correspondences between cells. All losses are optimized for encoder E, decoder D, and the mapping matrix P.

## 3 Results

### 3.1 Datasets

To assess the accuracy and generalizability of our proposed mapping framework, we select spatial atlas and scRNA-seq datasets from three distinct biological systems: drosophila embryo (DE), mouse primary visual cortex (VISp), and human cancerous pancreas. As for the DE, the scRNA-seq data are from the GEO database with accession number GSE95025 (Karaiskos *et al.* 2017), and the spatial reference utilized in the experiments is acquired from the Berkeley Drosophila Transcription Network Project (BDNTP) (Larkin *et al.* 2021). In the case of the mouse VISp, the corresponding Smart-Seq dataset (Tasic *et al.* 2018) was used. The corresponding spatial atlas is obtained from various ST technologies, MERFISH, smFISH, STARmap, seqFISH+, and 10× Visium. seqFISH+ data are primarily used for generating synthetic data (Li *et al.* 2022a) to assist in the quantitative evaluation of spot deconvolution. Data download instructions are included in Supplementary.1 Data availability, available as supplementary data at *Bioinformatics* online. For the human biological system analysis, pancreatic ductal adenocarcinoma (PDAC) tissues, with a diameter extending to 100 μm, were selected for this study. Cell-type deconvolution were performed on 428 spatially distinct spots correlated with 1926 individual cells, encompassing the expression of 19,736 genes (Moncada *et al.* 2020). For a detailed dataset overview, please refer to Table 1.

**Table 1.** The detailed information of benchmarking datasets.^a^

BS	Dataset	Protocols	Cells/Spots	Genes
Drosophila Embryo	Spatial Atlas	FISH	3039	84
	scRNA-seq	Drop-seq	1297	8924
Mouse VISp	Spatial Atlas	STARmap	1549	1020
		MERFISH	2399	254
		smFISH	2340	22
		seqFISH+	524	10,001
		10X Visium	2695	32,285
	scRNA-seq	Smart-seq	15,413	45,768
PDAC	Spatial Atlas	microarray-based	428	19,736
	scRNA-seq	inDrop	1926	19,736

a “BS” denotes the biological system.

We adhere to standardized pre-processing procedures. Specifically, the imaging-based ST data was normalized to ensure uniform total cell counts. The scRNA-seq and sequencing-based ST data were normalized based on the total counts across all genes within each cell. They were then scaled by 10e4 and subjected to log transformation with the addition of a pseudo-count.

### 3.2 Evaluation

After the mapping is completed, annotations can be transferred either from ST data to scRNA-seq data or vice versa. To assess the accuracy of the matching algorithm, we calculate the similarity and discrepancy between the predicted spatial ST data and the actual ST data. The evaluation metrics include the Pearson Correlation Coefficient (PCC) for measuring correlation, Structural Similarity Index (SSIM) for evaluating structural similarity, and Root Mean Square Error (RMSE) and Jensen–Shannon Divergence (JSD) for quantifying the difference between predicted and actual data. Superior matching performance is indicated by higher PCC and SSIM values, coupled with lower RMSE and JSD scores. We used a k-fold cross-validation strategy to evaluate gene expression reconstruction. For spot deconvolution and cell-type localization, we derive the distribution matrix per location. More details can be found in Supplementary.2 Validation, available as supplementary data at *Bioinformatics* online.

### 3.3 Competing methods

We compare our proposed method against several state-of-the-art cell mapping methods: Tangram (Biancalani *et al.* 2021), novoSpaRc (Moriel *et al.* 2021), SpaOTsc (Cang and Nie 2020), and GraphST (Long *et al.* 2023). Tangram, novoSpaRc, and SpaOTsc were chosen due to their widespread adoption, while GraphST represents a recently proposed deep-learning approach using graph neural networks.

### 3.4 Spatial gene reconstruction

**Figure 2.**
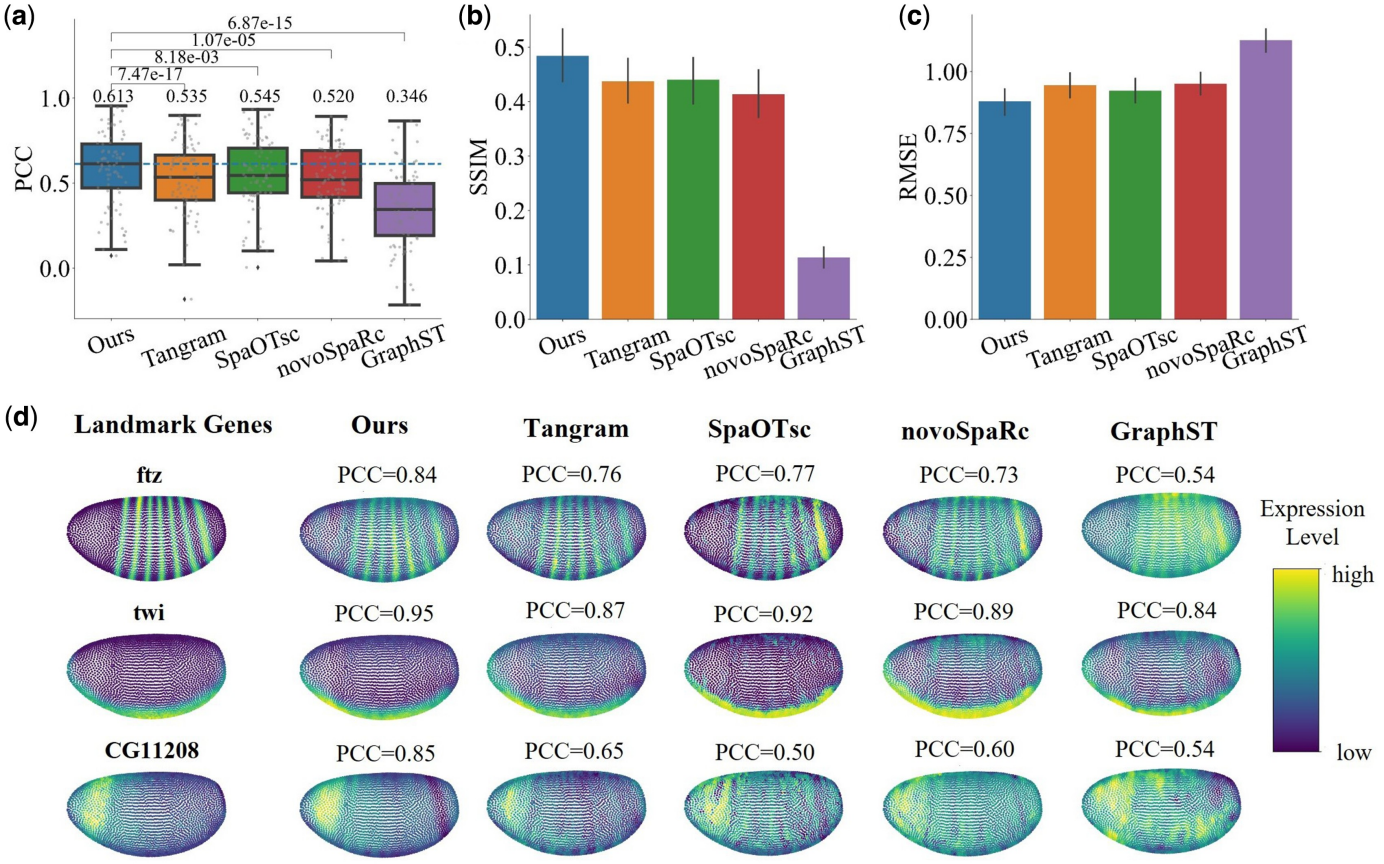
The performance of spatial gene reconstruction of single-cell DE data. (a) PCC of different methods. The Wilcoxon signed-rank test and the median of PCC are reported. (b) SSIM of different methods. (c) RMSE of different methods. The bar chart represents the mean values of the data, with error bars indicating the ± 95% confidence intervals. (d) Predicted expression of test genes. The reconstruction results show the genetic patterning of the reconstructed scRNA-seq of different methods and their corresponding atlas (ground truth). The highlighted portions represent genes with high expression and vice versa.

Due to the high-throughput nature of scRNA-seq, we can detect more gene expressions at the single-cell level. Mapping scRNA-seq data onto atlases helps us to understand the spatial patterns of the extended genes, which are not on the atlas. To evaluate the effectiveness of the mapping results in predicting spatial gene expression, we first applied COME to the spatial reconstruction of DE data. Initially, we illustrated the distribution of the PCC between each gene’s actual and predicted spatial expression profiles. For each comparative analysis, one-sided paired Wilcoxon signed-rank tests are performed to ascertain whether COME yields significantly higher PCC values than baseline methods. The results in Fig. 2a demonstrate that COME significantly surpasses the performance of the leading alternative, SpaOTsc, and markedly enhances the median PCC relative to other evaluated methods (with *P*-value < .05). In addition, COME consistently and significantly outperformed the PCC, SSIM, and RMSE compared to other methods on DE data. From Supplementary Fig. S1, available as supplementary data at *Bioinformatics* online, it can be observed that the coefficients learned by COME align with biological expectations. Furthermore, from Supplementary Fig. S2, available as supplementary data at *Bioinformatics* online, it can be seen that COME helps to remove batch effects between ST and scRNA-seq.

We further select three typical spatial landmark genes (Karaiskos *et al.* 2017, Miller *et al.* 2021) from DE and visually compare the predicted spatial patterns with their respective ground truth and other methods (as shown in Fig. 2d). A higher PCC signifies a more successful reconstruction. The results demonstrate the successful reconstruction of some well-characterized genes to show that our reconstruction method is feasible. More specifically, in drosophila embryos, the expression of the gene *twi* is typically observed in the ventral cells during the blastoderm stage (Ashraf *et al.* 1999). We observe that gene *ftz* reconstruction exhibits distinct spatial features, with only six stripes distributed within the tissue. Gene *CG11208* reconstruction is only shown on the drosophila’s dorsal, and the PCC is significantly improved when compared with the results of other methods. These three representative genes have clearer outlines than other methods and are more consistent with the ground truth.

### 3.5 Evaluation on cellular resolution spatial transcriptomic data

**Figure 3.**
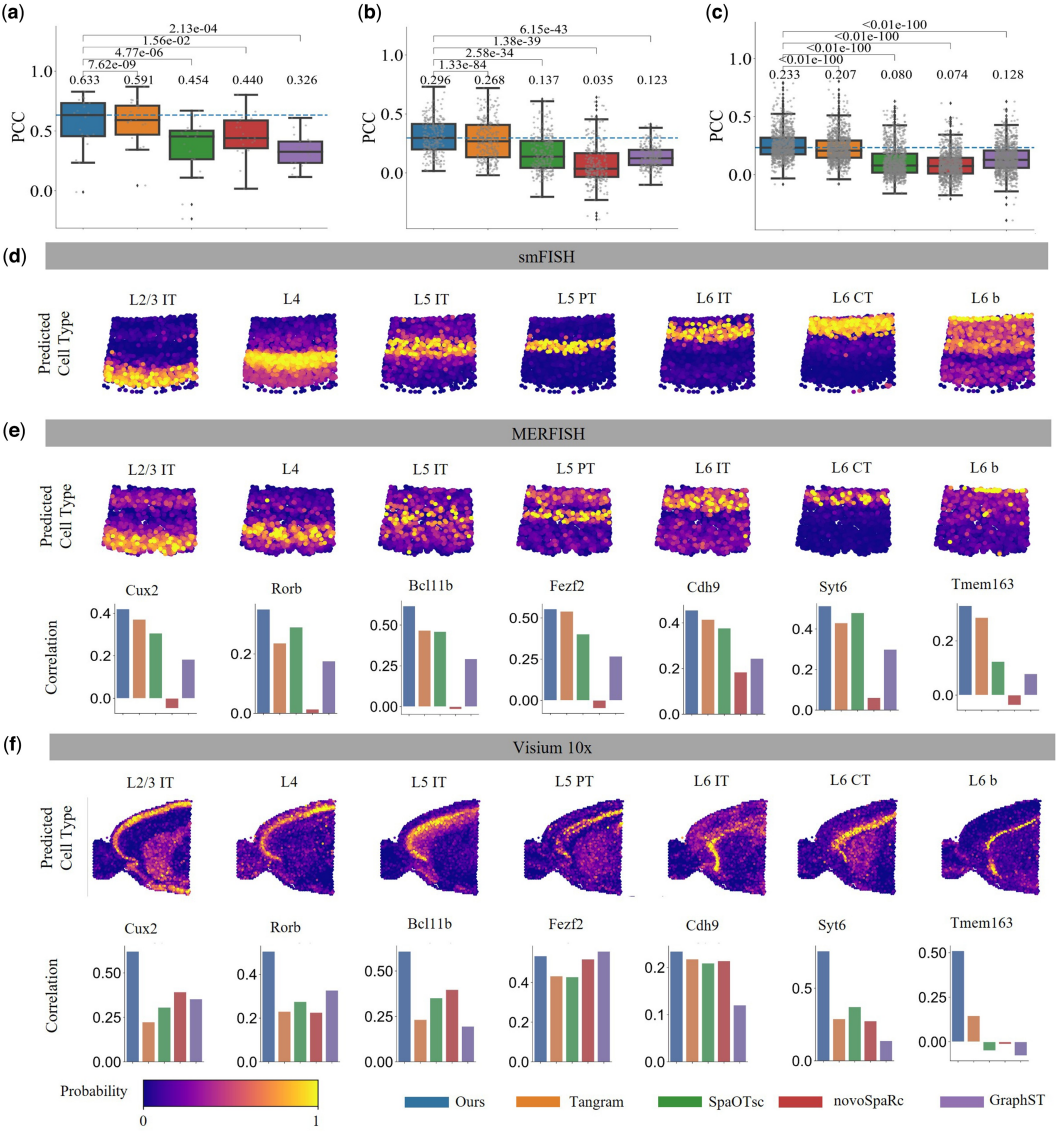
Performance of COME on the mouse VISp dataset from Smart-seq. (a) PCC of predicting gene spatial patterns on smFISH compared with different methods. (b) PCC of predicting gene spatial patterns on MERFISH compared with different methods. (c) PCC of predicting gene spatial patterns on STARmap compared with different methods. (d) Probabilistic spatial localization of scRNA-seq data on smFISH reference. (e) Probabilistic spatial localization of scRNA-seq data on MERFISH reference. In the first row, the inferred probabilities of the seven glutamatergic neuron types by COME are displayed for each spatial spot. The next row presents a comparative analysis of the PCC between the predicted expression levels and marker genes across different methods. (f) Probabilistic spatial localization of scRNA-seq data on 10× Visium reference. In the first row, the probabilities of the seven glutamatergic neuron types inferred by COME are displayed on each spot. The next row presents a comparative analysis of the PCC between the predicted expression levels and marker genes across different methods.

Here, we apply our method to infer genes and localize cell types on a large scale for both cellular and non-cellular resolution ST data of mouse VISp generated from Smart-seq. As shown in Fig. 3a–c, the gene prediction results indicate that COME surpasses all competing methods in terms of PCC across all datasets. Notably, on the STARmap dataset, the performance of COME is particularly remarkable, with a median PCC value of 0.233. This represents a significant improvement of 12% compared to the second-best Tangram method. It is worth noting that the available cell gene counts for MERFISH, and STARmap datasets are denoted as 254 and 996 each. The network trained on these datasets, focusing on gene expression in cells with higher complexity, exhibits a clear advantage. This suggests that our proposed method excels at capturing intricate correlations in gene expression within complex cells, likely due to more accurate modeling of intercellular information.

Since most atlases have coarser cell-type resolution than the scRNA-seq data, our accurate mapping can potentially lead to a better understanding of how cell types are spatially distributed within the tissue. The cerebral cortex is mainly divided into three major categories: glutamatergic neurons, GABAergic neurons, and non-neuronal cells. Among them, glutamatergic neurons are cells with distinct spatial features, meaning that the distribution of glutamatergic neuron cells, such as “L2/3 IT”, “L4”, “L5 IT”, “L5 PT”, “L6 IT”, “L6 CT”, and “L6 b” in the tissue should exhibit clear stratification. Our mapping results (Fig. 3d–f, and Supplementary Fig. S3, available as supplementary data at *Bioinformatics* online) show that scRNA-seq data mapped to different atlases show spatially layered cells in glutamatergic neurons. In the mouse VISp, different genes exhibit higher expression in different layers (Alon *et al.* 2021, Cheng *et al.* 2022, Mao and Staiger 2024). For the image-based ST data, the visualization results in Supplementary Fig. S4, available as supplementary data at *Bioinformatics* online, of marker gene and gene reconstruction are explained for their consistency. According to Supplementary Fig. S4, available as supplementary data at *Bioinformatics* online, we can see that the spatial pattern of genes predicted by smFISH based on the mapping method is consistent with the marker gene atlas of MERFISH. For the Visium data, our method successfully reconstructs clear stratification from layer 2 to layer 6, as shown in Fig. 3f, consistent with previous studies (Wei *et al.* 2022, Wan *et al.* 2023). Our predicted results are also closer to the ground truth than other methods (Fig. 3e and f), suggesting that the predicted marker gene expression more strongly supports cell-type proportion inferred by COME. It should be highlighted that the spatial distribution of the subtypes within layer 6 is difficult to distinguish in a stratified manner. For instance, the characteristic gene of “L6 b”, *Tmem163*, unlike genes localized exclusively to particular areas, is distributed more diffusely across various layers yet predominantly accumulates at the top edges of layer 6. This distribution of “L6 b” deduced from COME aligns closely with analyses (Mao and Staiger 2024). Similarly, since IT neurons require multiple marker genes to ascertain the cell type (Fischer and Gillis 2021), the more accurate spatial distribution of scRNA-seq genes predicted using COME subsequently aids in inferring the distribution of IT neurons, which primarily within the more medial regions of the layer (Zhang *et al.* 2021), the comparison between the predicted results for “L5 IT” and “L5 PT” and those from Tangram can also be observed in Supplementary Fig. S5, available as supplementary data at *Bioinformatics* online.

Overall, our mapping method indicates that COME can recover spatial patterns for genes absent from the ST data and can further help understand the spatial distribution of cell types in tissues.

### 3.6 Spatial deconvolution

To validate the performance of COME, we use synthetic seqFISH+ ST dataset for quantitative analysis of the mapping results, and also conducted qualitative analysis using a real-world dataset (PDAC). From Supplementary Fig. S8, available as supplementary data at *Bioinformatics* online, compared to the second-best mapping method, SpaOTsc, our method achieves better results in SSIM and JSD, while maintaining relatively small variances in PCC and RMSE, indicating robust performance. Notably, the incorporation of prior knowledge further enhances the effectiveness of our method (COME+), allowing for competitive performance in downstream tasks, illustrating its versatility and adaptability in various applications.

**Figure 4.**
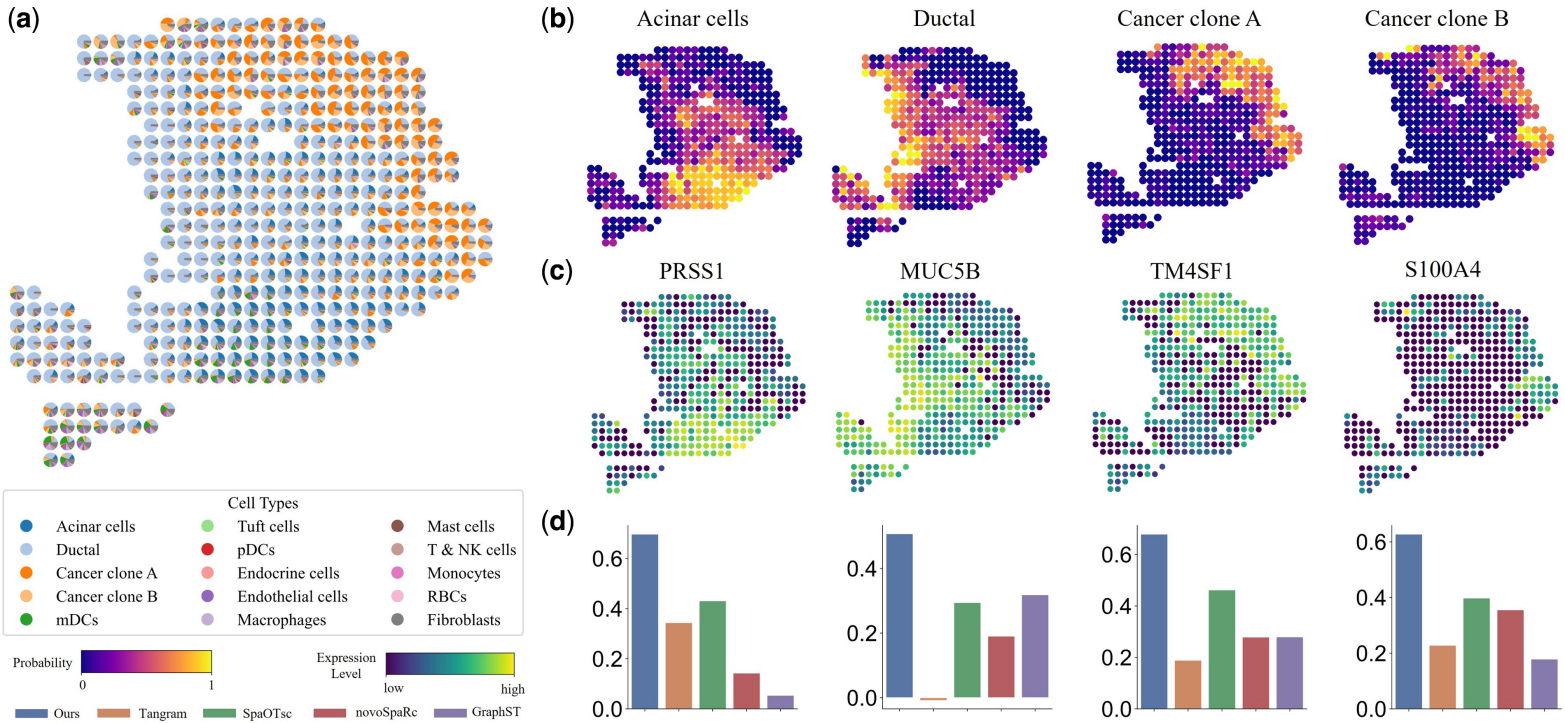
Spatial deconvolution result of PDAC. (a) Visualization of the spot deconvolution results. A spatial scatter pie chart is presented, which depicts the cell-type compositions as predicted by the COME. Each individual scatter point on the plot represents a distinct spatial spot within the ST data. (b) Predicted distribution of typical clusters in PDAC. (c) Expression profiles of marker genes corresponding to the cell clusters depicted in (b). (d) The comparison of the PCC between the inferred expression levels and marker genes with other methods.

We utilized the mapping estimated by COME to perform spot deconvolution across 15 cell types in actual human PDAC ST data. As shown in Fig. 4a, the result demonstrates the distinction between cancerous and non-cancerous areas throughout the region: tumor cell types are predominantly in cancerous areas (Orange). In contrast, normal pancreatic cell types are primarily found in non-tumor areas (Blue), consistent with previous studies’ results (Moncada *et al.* 2020). In comparison, other methods, such as Tangram and GraphST, do not delineate a separation between cancerous and non-cancerous areas (see Supplementary Fig. S6, available as supplementary data at *Bioinformatics* online). Additionally, COME can distinguish between ductal epithelium (light blue) and normal pancreatic tissue (dark blue), a differentiation that other mapping methods like SpaOTsc and novoSpaRc struggle to make (see Supplementary Fig. S6, available as supplementary data at *Bioinformatics* online).

Moreover, COME can accurately predict the four main cell-type positions of essential tumor microenvironment (TME) constituents, including ductal and acinar cells and cancer clones A and B, as shown in Fig. 4b. Cancer cell clones A and B are localized in two subregions of the cancerous area, with clone A predominantly occupying the top subregion and clone B predominantly distributed in the lower subregion. These findings align with the actual spatial profiles of the marker genes *TM4SF1* and *S000A4*. Their marker genes indicate accuracy in Fig. 4c and d demonstrates that COME outperforms other methods in the consistency of predicting marker genes by the PCC. Similarly, the cell subtype predictions (as shown in Supplementary Fig. S7, available as supplementary data at *Bioinformatics* online) for the TME in PDAC also conform to the previous study (Moncada *et al.* 2020).

## 4 Discussion

The essence of recovering the inherent spatial properties within scRNA-seq datasets lies in learning the cell-to-spot relationships between scRNA-seq and different spatial transcriptomics data. In this study, we introduce a mapping method for the spatial reconstruction of scRNA-seq data to facilitate knowledge transfer. Our approach integrates mapping with structural similarity regularization to leverage cell-spot dependencies. Additionally, we incorporate a contrastive learning strategy to enhance spatial awareness and distinguish between similar cell types within the latent feature representations of both modalities. The experimental results demonstrate that the proposed method accurately infers spatial information for scRNA-seq through the learned cell-to-spot mapping. More importantly, we provide biological insights to verify the effectiveness and practical significance of our proposed approach.

## Supplementary Material

btaf083_Supplementary_Data

## Data Availability

The data underlying this article are available in Zenodo Repository, at 10.5281/zenodo.14695533.
