# Supplementary material for: COME: contrastive mapping learning for spatial reconstruction of single-cell RNA sequencing data
Source: Bioinformatics. 2025 Feb 24;41(3):btaf083. doi: 10.1093/bioinformatics/btaf083 (PMC11897431; doi:10.1093/bioinformatics/btaf083)
Supplement: btaf083_Supplementary_Data [file btaf083_supplementary_data.pdf]

Supplementary

Supplementary.1 Data availability

Data for smFISH and MERFISH VISp are accessible at The SpaceTx Consortium (https://spacetx.github.io/data.html). For the Visium VISp dataset, we selected the anterior section from the 10x Genomics Data Repository (https://www.10xgenomics.com/resources/datasets), which comprises 2,695 spots and 32,285 genes. The sythetic seqFISH+ ST data are obtain form https://github.com/QuKunLab/SpatialBenchmarking. Additionally, the STARmap dataset is publicly available, as referenced in (Wang *et al.*, 2018).

Supplementary.2 Validation

Gene reconstruction: Concerning spatial reconstruction of gene expression, by utilizing $\mathbf{P}$, we can generate a predicted spatial gene expression matrix $\mathbf{G} = \mathbf{P}\mathbf{X}_{sc}$. This matrix indicates gene distribution and expression levels across the entire tissue. Comparing the predicted spatial gene expression matrix with the actual gene atlas allows us to evaluate the accuracy of our reconstruction. We evaluate the spatial gene reconstruction by the PCC, SSIM and RMSE. These metrics provide a comprehensive quantitative evaluation of the similarity between the predicted and true spatial gene expression patterns.

We employ the k-fold cross-validation strategy for the gene imputation task as Tangram for training and testing on benchmark datasets. The dataset is initially partitioned into training and testing sets based on genes. Specifically, for the MERFISH dataset, we split the 254 marker genes into 229 training genes and 25 testing genes. The training process is reiterated $k = 10$ times, wherein each iteration excludes different sets of genes for testing, thereby providing predictions for each gene. Moreover, for the smFISH, STARmap, and Drosophila benchmarks, the value of k is also set to 10.

To prevent overfitting, the number of training epochs varies for different benchmarks: 70, 100, 150, and 200 for Drosophila, smFISH, MERFISH, and STARmap datasets, respectively. For optimizing the mapping matrix $\mathbf{P}$, we employ gradient descent using the Adam optimizer (Kingma and Ba, 2015), with a learning rate of $\eta = 0.001$ and momentum parameters $\beta_1 = 0.5$ and $\beta_2 = 0.999$. The hyperparameter λ in Eq. (4) is set to 0.001 and the temperature τ is set to 0.8. Across most benchmarks, we can obtain an effective solution for the optimization problem by adopting equal weighting factors for the loss terms without heavy tuning.

Spot Deconvolution and Cell-type Localization: To transfer cell types in scRNA-seq data based on their annotations, the 3000 most highly variable genes (HVGs) are chosen as inputs for the COME model. We encode the cell type information as a one-hot matrix $\mathbf{A} \in \mathbb{R}^{M \times N_{annot}}$, where there are N_{annot} cell types across M cells. Based on the probability matrix $\mathbf{P}$, we derive the per-location distribution matrix $\mathbf{A}^* = \mathbf{P}^{\mathbf{T}}\mathbf{A}$, which represents the distribution of each cell type across spatial locations in the atlas. The resulting matrix $\mathbf{A}^* \in \mathbb{R}^{N \times N_{annot}}$ represents the composition of N_{annot} cell types at each of the N spatial spots. For the single-cell resolution (imaging-based) ST, we localize the cell type of each single-cell location based on the row in matrix $\mathbf{A}^*$ with the highest probability. We calculate PCC, SSIM, RMSE, and JSD to compare the predictions with each spot's density using the simulated seqFISH+ dataset.

Supplementary.3 The effectiveness of proposed contrastive learning framework

The proposed contrastive learning framework in our method offers a significant advantage by distinguishing between cell types, particularly in the context of spatial location prediction. By incorporating cell type information, we leverage the biological insight that cells of the same type often exhibit similar expression patterns and share spatial proximity. This enables the model to focus on meaningful biological relationships and variations, rather than treating each cell independently.

Incorporating cell type information into the learning process enhances the model's ability to capture both cell-to-cell relationships and spatial dependencies. For example, cells of the same type are likely to be located in nearby or related spatial regions. Recognizing these cell-type similarities improves the accuracy of spatial predictions. By explicitly distinguishing between different cell types, the contrastive learning framework better aligns scRNA-seq data with spatial transcriptomics data, resulting in more accurate spatial location predictions and biologically meaningful mappings. This approach addresses the limitations of purely position-based methods, which often overlook functional relationships between cells, such as those defined by cell type.

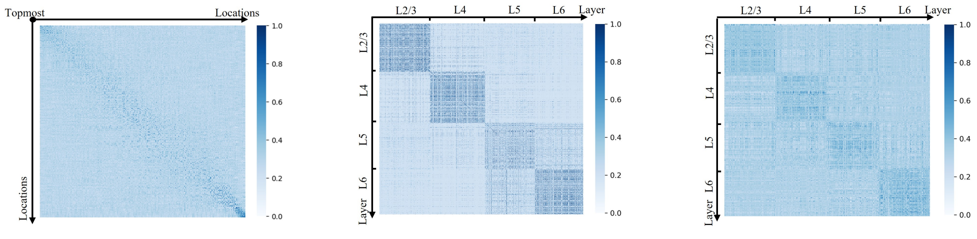

(a) Drosophila Embryo (b) Mouse Visual Cortex (smFISH) (c) Without cell-type contrastive learning

Fig. S1. Adaptive coefficient matrix $\mathbf{C}$ in two different biological systems. As analyzed in (Moriel *et al.*, 2021), non-cancer cells in physical proximity often share similar transcriptional profiles. To evaluate whether our method can capture such relationships, we input spatial transcriptomic (ST) data and employ COME network learning to construct our coefficient matrix $\mathbf{C}$. This matrix can reflect cell-spot relationships throughout the entire tissue. (a) The coefficient matrix $\mathbf{C}$ in Drosophila Embryo. Higher expression values are observed around the diagonal in the Drosophila Embryo tissue, and the expression level decreases gradually with increasing distance. (b) The coefficient matrix $\mathbf{C}$ in the mouse visual cortex cells. We sort cells in ST data (smFISH) into different layers to explore whether the learned coefficient within the same layer exhibits similar expression patterns. The learned coefficient matrix clearly displays four blocks on the diagonal, which is in accordance with the four cortical layers. The inferred patterns demonstrate that our networks can capture cell-spot gene expression relationships. (c) The coefficient matrix $\mathbf{C}$ training without cell-type contrastive learning. In (c), the coefficient $\mathbf{C}$ learned without incorporating cell-type information is more dispersed and does not form as clear diagonal blocks as seen in (b).

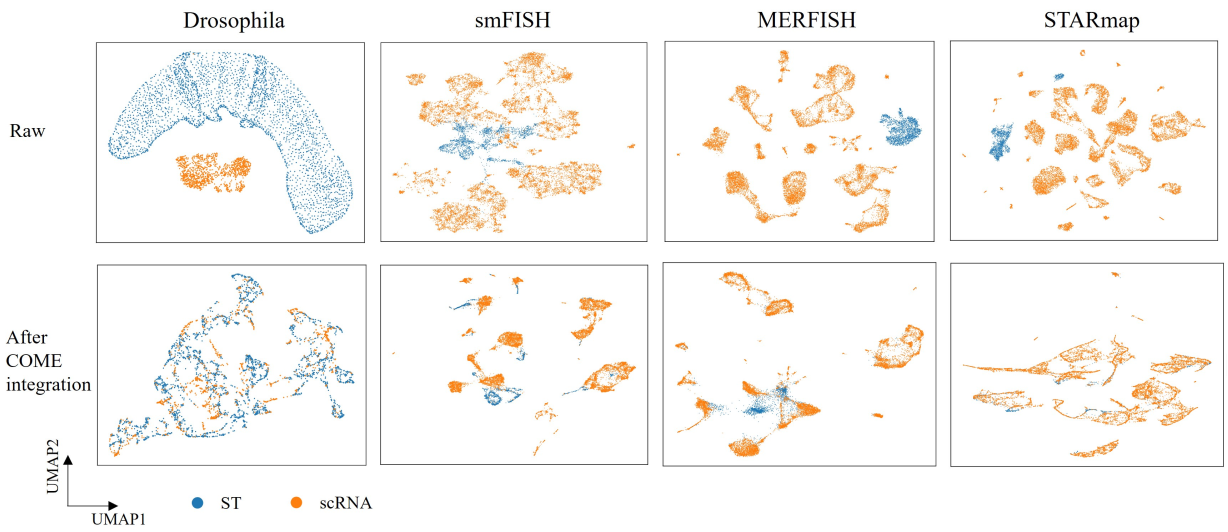

Fig. S2. Uniform Manifold Approximation and Projection (UMAP) visualization of imaging-based ST and scRNA paired data before integration and after COME integration. Before integration, both ST data and scRNA data exhibit batch effects, which can be clearly distinguished in UMAP visualization. After COME integration, COME removes the batch effect, and the paired data are visibly clustered.

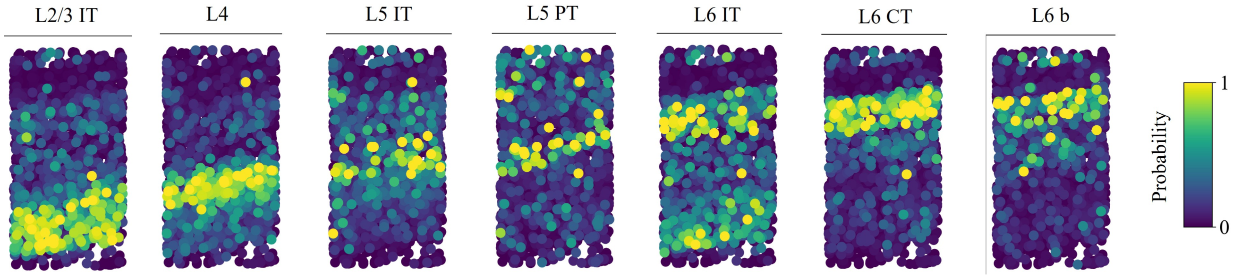

Fig. S3. Probabilistic assignment of spots to scRNA-seq data on STARmap reference. The predicted locations of neuronal cell types in the Smart-seq dataset similarly exhibit a layered growth pattern on the STARmap atlas.

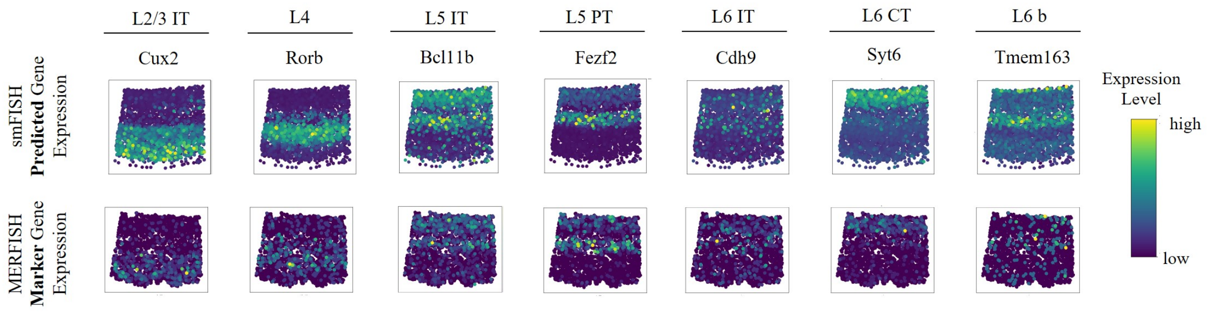

Fig. S4. The performance of spatial gene reconstruction of single-cell Mouse VISp data. The first row shows smFISH extended gene spatial patterns inferred by COME. The next row exhibits the expression levels of the corresponding MERFISH seven marker genes.

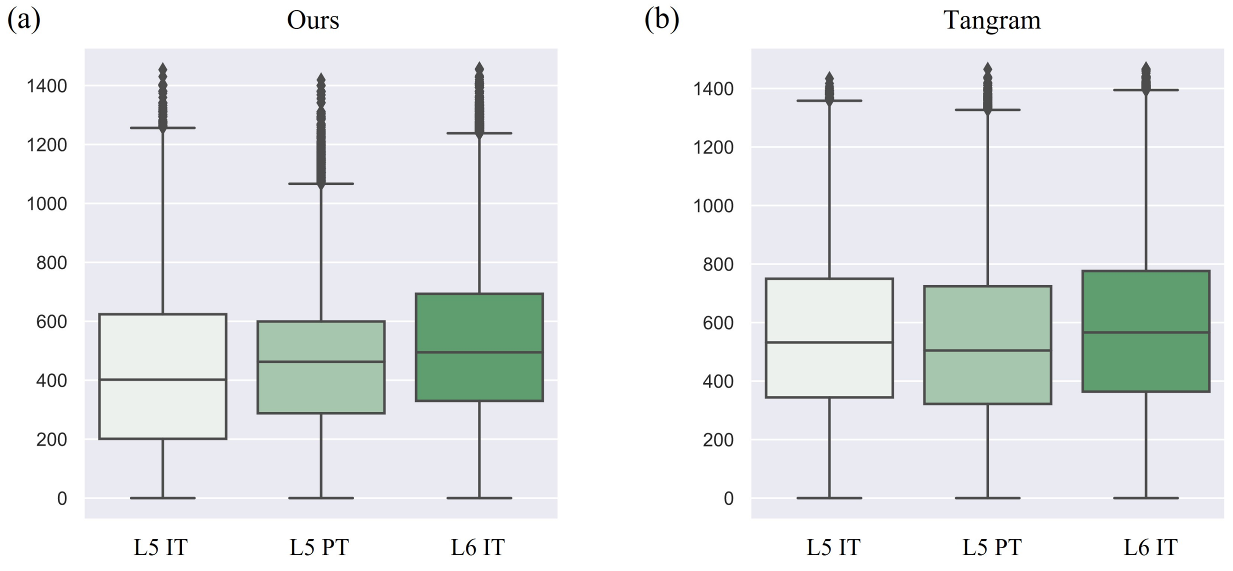

Fig. S5. Relative distance between cells from *L5 IT*, *L5 PT* and *L6 IT* in MERFISH. Data are presented as boxplots (minima, 25th percentile, median, 75th percentile, and maxima). (a) The relative distance between cells of our prediction. (b) The relative distance between cells of Tangram's Prediction. Within the cortical organization, *L5 PT* in Layer 5 neurons are generally situated closer to Layer 6 compared to *L5 IT* neurons. This is because *L5 PT* neurons typically occupy the deeper regions of Layer 5, adjacent to the border with Layer 6. Their long axons often project to subcortical areas, such as the brainstem and spinal cord. In contrast, *L5 IT* neurons are positioned in the upper or middle part of Layer 5, making *L5 PT* neurons relatively closer to L6. In (a), our predictive results suggest a gradual increase in the positioning of *L5 IT*, *L5 PT*, and *L6 IT* cells, whereas the arrangement in Tangram appears more uniform in (b).

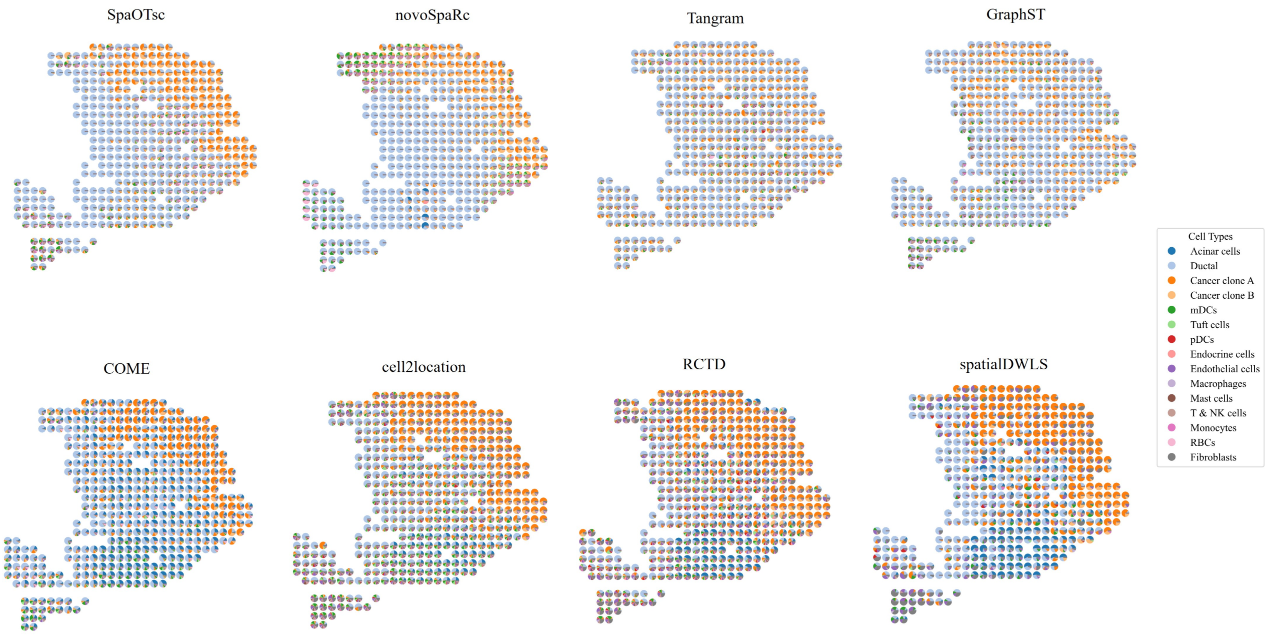

Fig. S6. The deconvolution results of the competing methods.Our predictions are more comparable to those of cell2location, RCTD, and spatialDWLS, and they surpass other mapping methods by clearly distinguishing key tissue types, such as ductal epithelium (light blue), normal pancreatic tissue (dark blue), and cancer clones (orange).

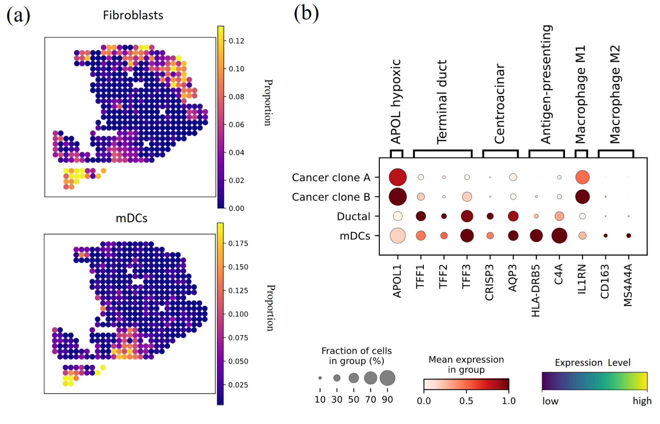

Fig. S7. The deconvolution results and analysis of TME. (a) Prediction of *fibroblast* and *mDCs* distribution. The results are consistent with the cancer-associated fibroblasts (CAF) phenomenon: Fibroblasts within the TME promote tumorigenic features by remodeling the extracellular matrix or secreting cytokines. *mDCs* are predicted on the bottom of the tissue. (b)The dotplot of the predicted ductal and macrophage subpopulation. The top of the dotplot indicates the subtypes of ductal and macrophage cells, while the bottom shows the corresponding marker genes. The four predicted cell types on the left side indicate four zones according to the localized cell type outlined in Fig. 4a and Fig. S7a. For instance, cancer leads to cell hypoxia, and the predicted ductal subtype *APOL hypoxic* corresponds to the area where the Cancer clone is located. Notably, the predicted *M1* is present in the Cancer Region, whereas *M2* is not. (c) The ground truth of the distribution of the marker genes in ductal and macrophage subpopulations. From (c), we can discern the accuracy of the predictions in (a) and (b). The spatial pattern of the corresponding marker gene *APOL1* is also distributed in the cancer region. *Terminal ductal* and *centroacinar* distributions are in the ductal area, with corresponding distributions for *TFF3* and *CRISP3*. Besides being present in ductal cells, antigen-presenting tends to be more prevalent in mDCs, where it plays an immunological role. This is consistent with the spatial distribution of the *C4A*. The earliest sensitive marker of anti-inflammatory *IL1RN* is found in the cancer region, while *MS4A4A* is only minimally present in the macrophage area.

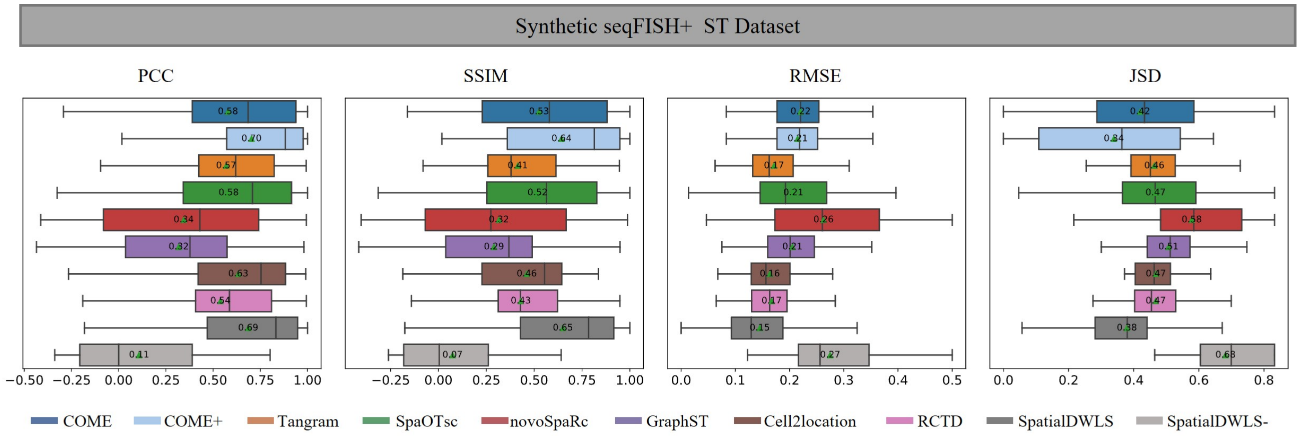

Fig. S8. Boxplots of PCC, SSIM, RMSE, and JSD values for the spot deconvolution simulated from seqFISH+ dataset. The average values are reported. The specialized spot deconvolution methods—Cell2location, RCTD, and SpatialDWLS—differ fundamentally from mapping-based approaches, as they incorporate prior knowledge in the form of gene expression signatures for each cell type (denoted as the signature expression matrix $\mathbb{S}$) derived from scRNA-seq data. Specifically, Cell2location employs a Bayesian framework to infer $\mathbb{S}$, SpatialDWLS utilizes enrichment analysis to obtain $\mathbb{S}$, and RCTD calculates $\mathbb{S}$ using a simple averaging strategy. To investigate the impact of such prior knowledge, we conducted additional experiments by integrating an enrichment-based prior (similar to SpatialDWLS) into our mapping framework. In this modified version, termed COME+, we adopt the enrichment analysis module from SpatialDWLS as prior knowledge while retaining mapping as the core component of the spot deconvolution process. Additionally, we define SpatialDWLS-, a variant of SpatialDWLS where the prior knowledge component $\mathbb{S}$ is removed. Our method achieves results comparable to those of specialized spot deconvolution methods such as cell2location, RCTD, and spatialDWLS. In particular, COME+ achieves competitive performance, demonstrating that supplementing mapping with additional prior knowledge improves spot deconvolution accuracy. Conversely, when prior knowledge is removed (SpatialDWLS-), performance decreases significantly. These results indicate the crucial role of cell type-specific expression signatures in spot deconvolution and highlight that our method can achieve strong performance when such prior knowledge is incorporated. Furthermore, our primary objective is to achieve accurate cell-to-spot mapping, which serves as a foundation for various downstream analyses. While COME functions effectively as a standalone framework for mapping and fundamental tasks such as spot deconvolution, its flexible architecture allows seamless integration of additional information (e.g., cell type signatures) to enhance specific downstream applications. This adaptability demonstrates that COME not only provides a comprehensive solution within a unified framework but also achieves superior performance when combined with task-specific prior knowledge.

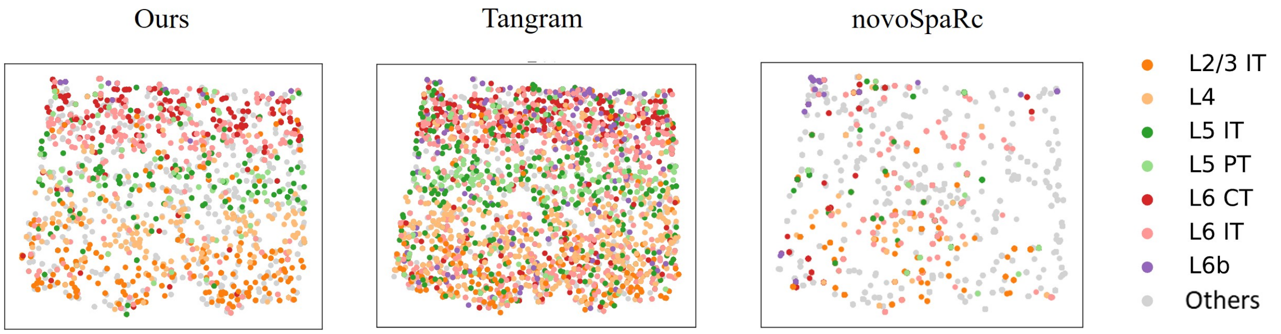

Fig. S9. Deterministic neurons' cell type prediction for MERFISH. The results demonstrate that our method achieves a more distinct localization of different cell types into separate layers compared to Tangram and novoSpaRc. This clearer separation aligns with biological expectations, where cells of the same type tend to cluster in adjacent tissue regions.

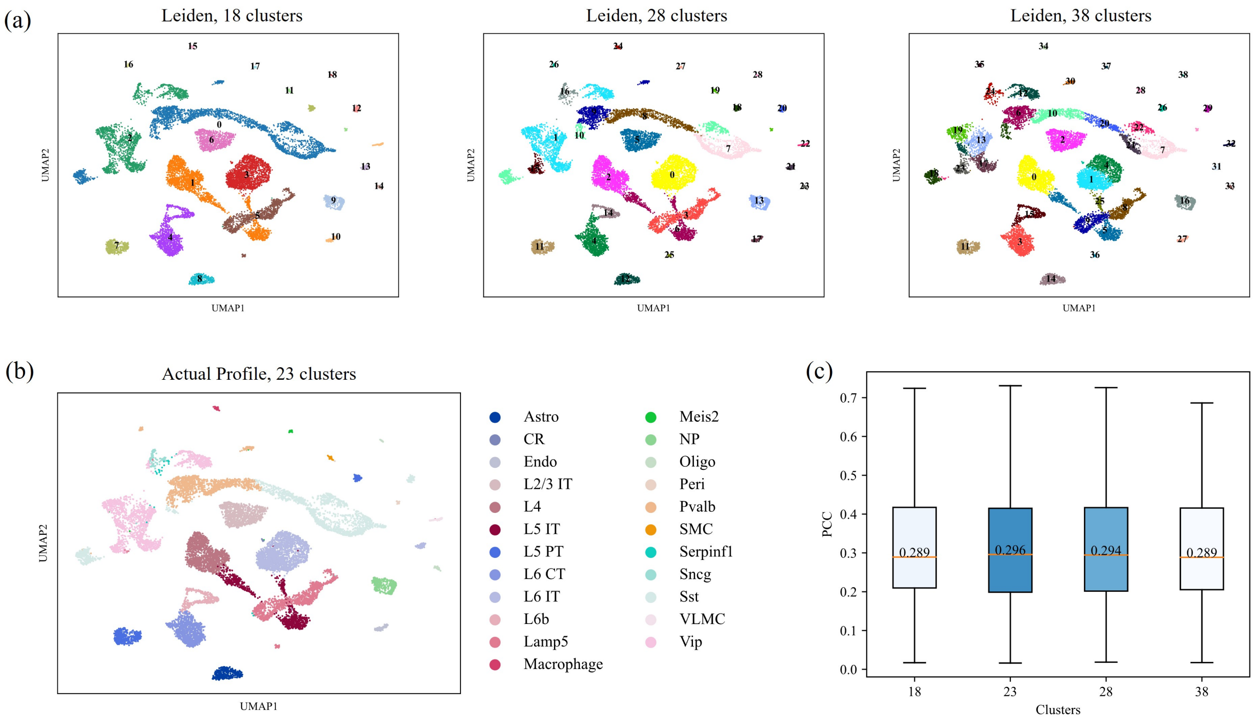

Fig. S10. (a) UMAP by Leiden with different resolution parameters for scRNA-seq, resulting in different numbers of cluster classifications. (b) UMAP of actual 23 different cell types. (c) PCC of gene reconstruction on different numbers of clusters. As long as the cell type annotations remain within a reasonable range, they do not introduce significant uncertainty into the experimental outcomes, demonstrating the robustness of our method. A common limitation of contrastive learning is its sensitivity to noisy labels, which can lead the model to learn incorrect features. However, in practice, our method demonstrates robust performance even in the presence of some noisy labels. Additionally, we evaluate our method using clustering-based cell type assignments instead of ground-truth labels, and it still achieves satisfactory performance,

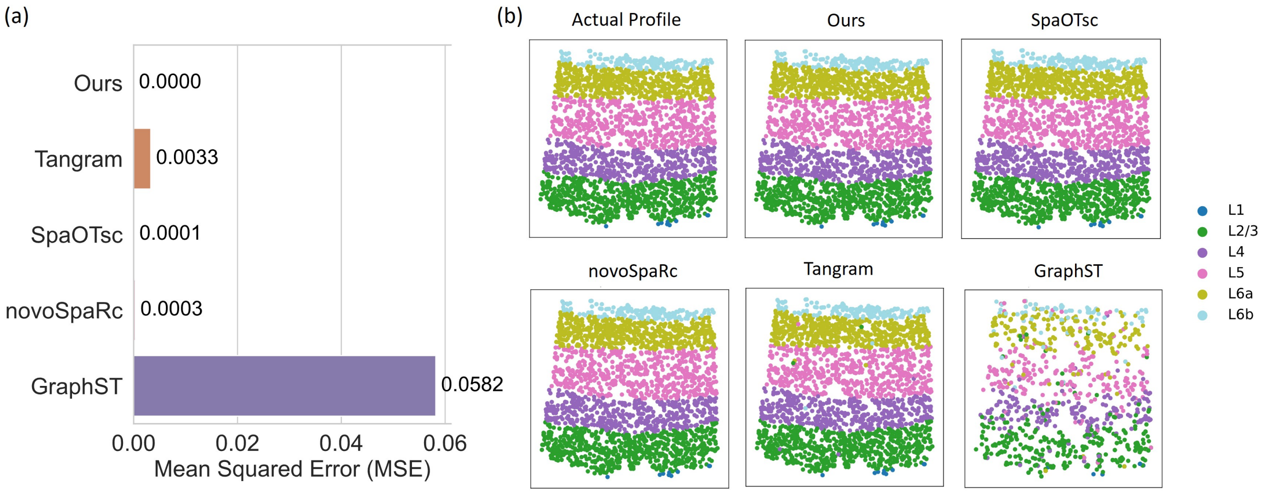

Fig. S11. Comparison of predicting single-cell resolution ST data. (a) The Mean Square Error (MSE) between predicted and real spatial locations across different methods; (b) The visualization of spatial predictions from five different methods. We conducted additional experiments using masked spatial locations in the MERFISH data as input to the scRNA-seq to evaluate the spatial reconstruction capability. These results indicate that COME, SpaOTsc, and novoSpaRc accurately reconstruct spatial locations, whereas GraphST struggles to preserve the original tissue structure. Additionally, Tangram exhibits minor discrepancies in predicting cell positions across different layers. Overall, these findings demonstrate COME's capability in predicting spatial locations.
